# Age-dependent immune heterogeneity and the limited diagnostic utility of peripheral blood biomarkers in pediatric tuberculosis: a real-world cohort study with multidimensional model evaluation

**DOI:** 10.3389/fimmu.2026.1954622

**Published:** 2026-10-07

**Authors:** Aifeng Liu, Jun Deng, Hongmei Chen, Xiaohua Ma

**Affiliations:** 1 Department of Pathology, The Affiliated Changsha Central Hospital, Hengyang Medical School, University of South China, Changsha, Hunan, China; 2 Department of Laboratory, The Affiliated Changsha Central Hospital, Hengyang Medical School, University of South China, Changsha, Hunan, China; 3 Department of Laboratory Medicine, The Second Medical College of Xinjiang, Karamay, Xinjiang, China

**Keywords:** age stratification, immune-inflammatory profile, lymphocyte subset, mycoplasma pneumoniae, pediatric tuberculosis, retrospective study

## Abstract

**Background:**

Pediatric tuberculosis (TB) remains a diagnostic challenge due to atypical presentations and low pathogen detection rates. Overlapping clinical features with *Mycoplasma pneumoniae* (MP) infection further complicate differential diagnosis. Immune-inflammatory parameters are age-dependent, yet how age modifies these profiles in children with TB and MP co-infection remains poorly understood.

**Objective:**

To investigate age-dependent heterogeneity in immune-inflammatory profiles among children with tuberculosis (TB) and to evaluate the additive diagnostic value of peripheral blood biomarkers beyond age alone, while assessing the impact of *Mycoplasma pneumoniae* co-infection on TB-related immune parameters.

**Methods:**

This retrospective observational study enrolled 280 children aged 0–18 years (186 TB cases, 94 non-TB controls) admitted between January 2024 and December 2025. Peripheral lymphocyte subsets, routine blood parameters, and systemic inflammatory indices (NLR, PLR, SII, MLR, SIRI) were compared between groups. Logistic regression identified independent correlates. ROC analysis, calibration curves, decision curve analysis (DCA), net reclassification improvement (NRI), and integrated discrimination improvement (IDI) were used to evaluate model performance. Age-stratified analysis (three strata: <5, 5–12, ≥12 years) was performed using ANCOVA with age as a covariate. Age × Group interaction analyses and 1:1 age-matched sensitivity analyses were conducted to formally test effect modification and exclude age confounding. Sensitivity analyses comparing bacteriologically confirmed versus clinically diagnosed cases and MP-only controls versus TB-MP co-infected cases were also performed.

**Results:**

Age was the sole variable independently associated with pediatric TB (adjusted OR = 1.118, 95% CI: 1.046–1.194, P = 0.001). TB patients exhibited significantly lower CD8^+^ T cell, NK cell, and lymphocyte counts and higher PLR compared with controls, but these differences were highly age-dependent. Age-stratified analysis revealed striking heterogeneity: immune-inflammatory differences were highly significant in adolescents ≥12 years (CD8^+^ T cells and NK cells: adjusted P < 0.009 and P < 0.008, respectively) but virtually absent in children <5 years (all P > 0.05). Formal Age × Group interaction analyses confirmed significant interactions for CD8^+^ T cells (P < 0.001, partial η^2^ = 0.089), NK cells (P < 0.001, partial η^2^ = 0.196), and lymphocyte count (P < 0.001, partial η^2^ = 0.404). Age-matched sensitivity analysis further confirmed that CD8^+^ T cell and NK cell differences in the ≥12 years stratum remained significant after matching (CD8^+^ T: P = 0.004; NK: P < 0.001), whereas CD4^+^ T cell differences were abolished (P = 0.113). The age-only model produced an AUC of 0.686 (95% CI: 0.618–0.755). The age + CD8^+^ T cell combined model showed statistically significant improvement (AUC difference = 0.041, 95% CI: 0.010–0.071; DeLong P = 0.009; likelihood-ratio test P < 0.001), with good calibration (Hosmer–Lemeshow P = 0.228; Brier score = 0.187; McFadden R^2^ = 0.127), positive net benefit across threshold probabilities of approximately 0.40–0.78 in DCA, and significant reclassification improvement (continuous NRI = 0.505, 95% CI: 0.258–0.755, P < 0.001; IDI = 0.038, 95% CI: 0.011–0.068, P = 0.003; event NRI^+^ = 0.419). None of the other age-combined models (age + NLR, SII, PLR, or MP) showed significant improvement over age alone (all DeLong P > 0.05). Within the TB cohort, MP co-infection was not observed to significantly alter immune-inflammatory profiles after age adjustment (all P > 0.05, partial η^2^ < 0.01). Sensitivity analyses confirmed immunological consistency between bacteriologically confirmed and clinically diagnosed cases (all P > 0.05, partial η^2^ ≤ 0.017).

**Conclusion:**

Age is the key variable associated with pediatric TB and demonstrates marked age-dependent immune-inflammatory heterogeneity. Peripheral blood immune biomarkers added modest diagnostic information beyond age alone, with statistically significant yet clinically modest improvements. The incremental value of CD8^+^ T cell count was most evident in intermediate-risk decision scenarios (threshold probabilities 0.40–0.78) and in adolescents ≥12 years, rather than as a universal screening tool. MP co-infection was not associated with significant alterations in TB-related immune-inflammatory profiles in this cohort. These findings emphasize age stratification as a core principle in pediatric TB evaluation and suggest caution regarding over-reliance on routine lymphocyte subset testing for diagnostic purposes, particularly in younger children where clinical and radiological assessment remains paramount.

## 1 Introduction

Pediatric tuberculosis (TB) continues to pose a substantial global public health threat, especially in high-TB-burden countries, where its incidence and mortality rates remain elevated (1). The clinical presentation of pediatric TB is frequently atypical and insidious in progression (2), and the low positivity rate of etiological examinations significantly hinders early diagnosis, often leading to delayed treatment (3).

Among the diverse pathogens causing childhood infections, *Mycoplasma pneumoniae* (MP) infection is one of the most common causes of pediatric respiratory tract infections (4). Notably, some clinical manifestations and imaging features of MP infection overlap with those of TB, making differential diagnosis particularly challenging in both single infections and co-infections (5). The etiological spectrum of pediatric respiratory infections is also broad, encompassing common pathogens such as *respiratory syncytial virus*, *Haemophilus influenzae*, and *Streptococcus pneumoniae* (6). In pediatric TB cases complicated by co-infections, robust evidence and a clear understanding of these complex clinical scenarios remain insufficient.

Comparative studies on immune-inflammatory profiles between pediatric TB and MP infection are limited, particularly regarding whether nonspecific immune parameters, such as peripheral blood lymphocyte subsets and systemic inflammatory markers, exhibit characteristic alterations across different infection types. Critically, these immune markers undergo profound age-dependent changes during childhood, yet how age modifies TB-related immune-inflammatory patterns in real-world settings remains poorly understood. Additionally, the interactions among age, co-infections, and TB-related immune profiles have not been systematically investigated in pediatric populations.

To address these gaps, this retrospective study collected clinical and laboratory data from children with different infectious diseases. The primary objectives were: 1) to compare immune-inflammatory profiles between TB and non-TB infections while accounting for MP co-infection status; 2) to identify which immune-inflammatory parameters are independently associated with pediatric TB after adjusting for age; 3) to evaluate whether these parameters provide incremental diagnostic value beyond age alone; and 4) to explore age-dependent heterogeneity in immune-inflammatory responses through stratified analysis. We anticipate that our findings will provide practical evidence to refine the early detection and differential diagnosis of pediatric TB, particularly by highlighting the role of age as a critical stratification variable.

## 2 Materials and methods

### 2.1 Study design and participants

This retrospective observational study enrolled pediatric patients aged 0–18 years with infectious diseases who were hospitalized in the Pediatric Tuberculosis Department of Changsha Central Hospital from January 2024 to December 2025. Participants were stratified into two groups based on infection etiology: the TB group (TB-related infections) and the non-TB control group (non-TB infections). Collected baseline data included demographic information (age and sex), causative pathogens (*Mycoplasma pneumoniae* [MP], *Haemophilus influenzae*, *Streptococcus pneumoniae*, respiratory syncytial virus, and other common infectious pathogens), laboratory indicators (acid-fast staining, mycobacterial culture, and TB nucleic acid detection), and comorbidities including vitamin D deficiency.

In accordance with the World Health Organization consolidated guidelines on tuberculosis, Module 5: Management of tuberculosis in children and adolescents (7), the diagnosis of pediatric tuberculosis was established through an integrated approach incorporating clinical manifestations (persistent cough >2 weeks, unexplained fever, weight loss, and night sweats), characteristic chest radiographic findings (hilar and/or mediastinal lymphadenopathy, or parenchymal infiltrates), and bacteriological confirmation via acid-fast bacilli staining, mycobacterial culture, and TB-DNA nucleic acid testing.

For clinically diagnosed cases without bacteriological confirmation, the diagnosis was based on a comprehensive assessment that required at least two of the following three criteria (1): a history of close contact with a patient having tuberculosis (2); chest CT findings highly suggestive of primary pulmonary tuberculosis, including tree-in-bud nodules, lobar consolidation, or mediastinal/hilar lymphadenopathy with necrotic features; and (3) a positive tuberculin skin test (TST) with induration ≥10 mm in diameter or a positive T-SPOT.TB result. Additionally, all clinically diagnosed patients showed clinical and radiological improvement after receiving standard anti-tuberculosis treatment.

The non-TB control group was composed of symptomatic children who were hospitalized during the same study period and diagnosed with common respiratory pathogen infections, including Mycoplasma pneumoniae (MP; serological titer ≥1:160 or PCR-positive), *H.influenzae*, and *S. pneumoniae*, and *respiratory syncytial virus* based on etiological testing. All children in the non-TB control group met the following criteria: (1) chest imaging findings showed no features suggestive of tuberculosis (e.g., hilar/mediastinal lymphadenopathy, tree-in-bud signs, or cavitary lesions); (2) T-SPOT.TB assay was negative or tuberculin skin test (TST) induration was <5 mm; (3) no prior anti-tuberculosis treatment had been administered; and (4) there was no history of close contact with a tuberculosis patient. The inclusion of a heterogeneous non-TB control group comprising various respiratory infections was intended to reflect the real-world clinical scenario in which pediatric tuberculosis must be differentiated from common childhood infections with overlapping clinical features. To validate the appropriateness of pooling these diverse controls, we performed a sensitivity analysis restricted to MP-only controls compared with MP-TB cases. The results confirmed that the heterogeneity of the control group did not materially influence our primary conclusions. Patients with acquired immunodeficiency syndrome, malignant tumors, severe cardiovascular diseases, or other critical systemic disorders were excluded. All personal identifiable information was anonymized prior to data analysis, and the requirement for informed consent was waived. This study was approved by the Ethics Committee of Changsha Central Hospital (Approval No.: 2024 Medical Review No. 290).

### 2.2 Data collection and index calculation

#### 2.2.1 Routine blood and inflammatory indicators

Peripheral blood samples were collected at admission for routine blood examination using an automated hematology analyzer. Routinely detected blood parameters included neutrophil count (NEUT), lymphocyte count (LYM), monocyte count (MONO), and platelet count (PLT). Five composite immune-inflammatory indices were calculated as follows:

Neutrophil-to-Lymphocyte Ratio (NLR) = NEUT/LYM

Monocyte-to-Lymphocyte Ratio (MLR) = MONO/LYM

Systemic Immune-Inflammation Index (SII) = (PLT × NEUT)/LYM

Platelet-to-Lymphocyte Ratio (PLR) = PLT/LYM

Systemic Inflammation Response Index (SIRI) = (NEUT × MONO)/LYM

#### 2.2.2 Flow cytometric detection of lymphocyte subsets

Peripheral blood samples were collected in EDTA-K_2_ anticoagulant tubes and processed within 6 hours of collection. Lymphocyte subset analysis was performed using the Agilent NovoCyte flow cytometer (Agilent Technologies (Hangzhou) Co., Ltd.). The following commercially available reagent kit was used according to the manufacturer‘s instructions: the CD3/CD16⁺CD56/CD45/CD4/CD19/CD8 detection kit (Agilent Technologies). These kits contain premixed monoclonal antibodies (mouse anti-human), including CD3-FITC, CD8-APC-Cy7, CD4-PE-Cy7, CD16^+^CD56-PE, CD19-APC, and CD45-PerCP-Cy5.5, as well as a proprietary red blood cell lysis solution. The antibody panel was designed to identify the following lymphocyte subsets: CD3^+^CD4^+^ T helper cells, CD3^+^CD8^+^ cytotoxic T cells, CD3^-^CD19^+^ B lymphocytes, and CD3^-^CD16^+^CD56^+^ natural killer (NK) cells.

Briefly, 50 μL of anticoagulated whole blood was aliquoted into labeled 5 mL polystyrene tubes, followed by the addition of 20 μL of the antibody cocktail from the respective kit. After gentle vortexing, the samples were incubated in the dark at room temperature for 15 minutes. Subsequently, 450 μL of the proprietary red blood cell lysis solution (diluted according to the manufacturer’s recommended protocol) was added. The samples were gently vortexed and incubated in the dark at room temperature for an additional 15 minutes. Following lysis, the samples were gently vortexed and immediately acquired on the flow cytometer.

Flow cytometric data were acquired on a NovoCyte D2060R flow cytometer system (Agilent Technologies (Hangzhou) Co., Ltd.) equipped with two lasers (488 nm and 640 nm) and six fluorescence detectors, and operated with NovoExpress software. Instrument performance was verified using NovoCyte QC Particles (Agilent Technologies) according to the manufacturer’s recommendations. For each sample, a minimum of 5,000 events within the lymphocyte gate were acquired. Compensation was performed automatically using single-stained controls for each fluorochrome.

Data analysis was performed using NovoExpress™ software (Agilent Technologies). The gating strategy was as follows: lymphocytes were first identified based on their forward scatter (FSC) and side scatter (SSC) characteristics. Doublet discrimination was performed using FSC-area versus FSC-height to exclude cell aggregates. Subsequently, lymphocytes were further gated based on SSC and CD45^+^ expression. Within the viable lymphocyte gate, CD3^+^CD4^+^ cells were identified as T helper cells, CD3^+^CD8^+^ cells as cytotoxic T cells, CD3^-^CD16^+^CD56^+^ cells as NK cells, and CD3^-^CD19^+^ cells as B lymphocytes. The percentage of each subset was determined, and absolute counts (cells/μL) were calculated using the volumetric method according to the manufacturer’s instructions.

#### 2.2.3 Diagnosis of *Mycoplasma pneumoniae*

MP infection was diagnosed based on either of the following criteria: (1) a single serum MP antibody titer ≥1:160 (particle agglutination [PA] assay), or a ≥4-fold rise in MP antibody titer between acute and convalescent sera; or (2) positive MP-DNA testing of respiratory specimens (including nasopharyngeal swabs and sputum). MP serology was performed using the particle agglutination (PA) method according to the manufacturer’s instructions (Zhuhai Lizhu Reagent Co., Ltd.).

#### 2.2.4 Detection of serum 25(OH)D concentrations

Serum 25(OH)D concentrations were measured using the Kingsr 25-hydroxyvitamin D assay kit (latex immunoturbidimetric method, Beijing Strong Biotechnologies, Inc.) on a Hitachi 7600 Automatic Biochemical Analyzer (Hitachi, Tokyo, Japan) according to the manufacturer’s instructions. Vitamin D status was classified according to the Chinese Guidelines for Clinical Practice Related to Vitamin D Nutrition in Children (2022) (8): serum 25(OH)D < 30 nmol/L was defined as deficiency, 30–50 nmol/L as insufficiency, and >50–125 nmol/L as sufficiency. All measurements were performed in the same laboratory using the same method for all patients.

### 2.3 Quality control

Internal quality control procedures for hematological analysis and flow cytometry were well implemented throughout the study. All external quality assessment results from the National Health Commission of China and Hunan Provincial Clinical Laboratory Center met or exceeded qualified standards, ensuring the accuracy and reliability of all laboratory data.

### 2.4 Statistical analysis

The Shapiro–Wilk test was used to evaluate the normality of continuous variables. Data with non-normal distribution were presented as median (interquartile range [IQR]). For statistical comparison, the Mann–Whitney U test was applied for two-group analyses, and the Kruskal–Wallis test was used for multiple-group comparisons when appropriate.

Univariate and multivariate logistic regression analyses were conducted to screen independent factors associated with pediatric TB. Variables with P < 0.10 in univariate analysis were included in the multivariate regression model. The final model was adjusted for age, sex, lymphocyte count, and MP co-infection. Results were expressed as adjusted odds ratios (ORs) with 95% confidence intervals (CIs). Variables with moderate collinearity (Pearson’s r > 0.4) were excluded from simultaneous inclusion. Model fitness was assessed using the Hosmer–Lemeshow test and Nagelkerke R^2^.

Sensitivity analyses were performed to verify the robustness of the predictive model. Lymphocyte count was replaced with CD4^+^T-cell count or vitamin D deficiency status in separate multivariate models, with detailed results shown in **Supplementary Table S1**.

Within the TB cohort, analysis of covariance (ANCOVA) with age as a covariate was performed to compare immune-inflammatory parameters between children with isolated TB infection and those with TB-MP co-infection. Given the skewed distribution, all lymphocyte subset counts and inflammatory indices were natural log-transformed prior to statistical analysis. For parameters with zero values, a constant of 0.5 was added before log transformation. Partial eta-squared (partial η^2^) was calculated to quantify the magnitude of between-group differences. Effect size magnitudes were interpreted following established standard thresholds: negligible effect (<0.01), small effect (0.01–0.05), medium effect (0.06–0.13), and large effect (≥0.14) (9).

Receiver operating characteristic (ROC) curves were plotted for single age and combined diagnostic models (age + NLR, age + SII, age + PLR, age + MP infection, and age + CD8^+^ T cells). The area under the curve (AUC) was calculated to evaluate diagnostic efficacy with corresponding 95% CIs. DeLong’s test was used to compare AUCs to determine whether combined models yielded superior diagnostic performance compared with age alone.

Model performance was further evaluated beyond AUC using the following analyses: (1) Calibration plots using Hosmer-Lemeshow goodness-of-fit tests and Brier scores; (2) Decision curve analysis (DCA) following the method of Vickers and Elkin to calculate net benefit across a range of clinically relevant threshold probabilities; and (3) Net Reclassification Improvement (NRI) and Integrated Discrimination Improvement (IDI) to quantify the incremental predictive value of adding CD8^+^T cell count to age alone, with 95% confidence intervals estimated via bootstrap resampling (2000 iterations). The optimal cutoff value for each model was determined using the Youden index (sensitivity + specificity − 1), selecting the cutoff that maximized this index. Sensitivity, specificity, positive predictive value (PPV), and negative predictive value (NPV) with their 95% confidence intervals were estimated using the bootstrap method (1000 iterations).

To explore age-dependent heterogeneity in immune-inflammatory profiles, patients were stratified into three subgroups according to immune developmental characteristics: <5 years (immature immunity), 5–12 years (progressively maturing immunity), and ≥12 years (near-adult immune function). Immune-inflammatory parameters were compared between TB and non-TB groups in each stratum using the Mann–Whitney U test. Given the exploratory nature of the stratified analyses, no formal multiple comparison correction was applied; all subgroup results should be interpreted with caution and considered hypothesis-generating. To address the limited sample size in the <5 years stratum (TB: n=14, control: n=30), we performed a sensitivity power analysis using G*Power 3.1. For a two-sample independent t-test (two-tailed, α=0.05, power=0.80), we calculated the minimum detectable effect size (Cohen’s d) given the available sample sizes.

Age-matched sensitivity analysis was performed to further exclude age confounding. Each TB patient was matched to one non-TB control using nearest-neighbor matching without replacement, with a caliper of ±2 years based on age. Immune parameters were compared between matched pairs using the Wilcoxon signed-rank test. The matched analysis was additionally repeated in the subgroup of pairs with index age ≥12 years.

To assess the potential impact of misclassification bias, the TB group was divided into bacteriologically confirmed (AFB, culture, or TB-DNA positive) and clinically diagnosed (negative for all three but meeting clinical criteria) subgroups. ANCOVA with age as a covariate was used to compare immune parameters between the two subgroups. Results are presented as estimated marginal means with 95% confidence intervals, and P < 0.05 was considered statistically significant.

## 3 Results

### 3.1 Flow diagram of patient selection

The patient selection process is summarized in **Figure 1**.

**Figure 1:**
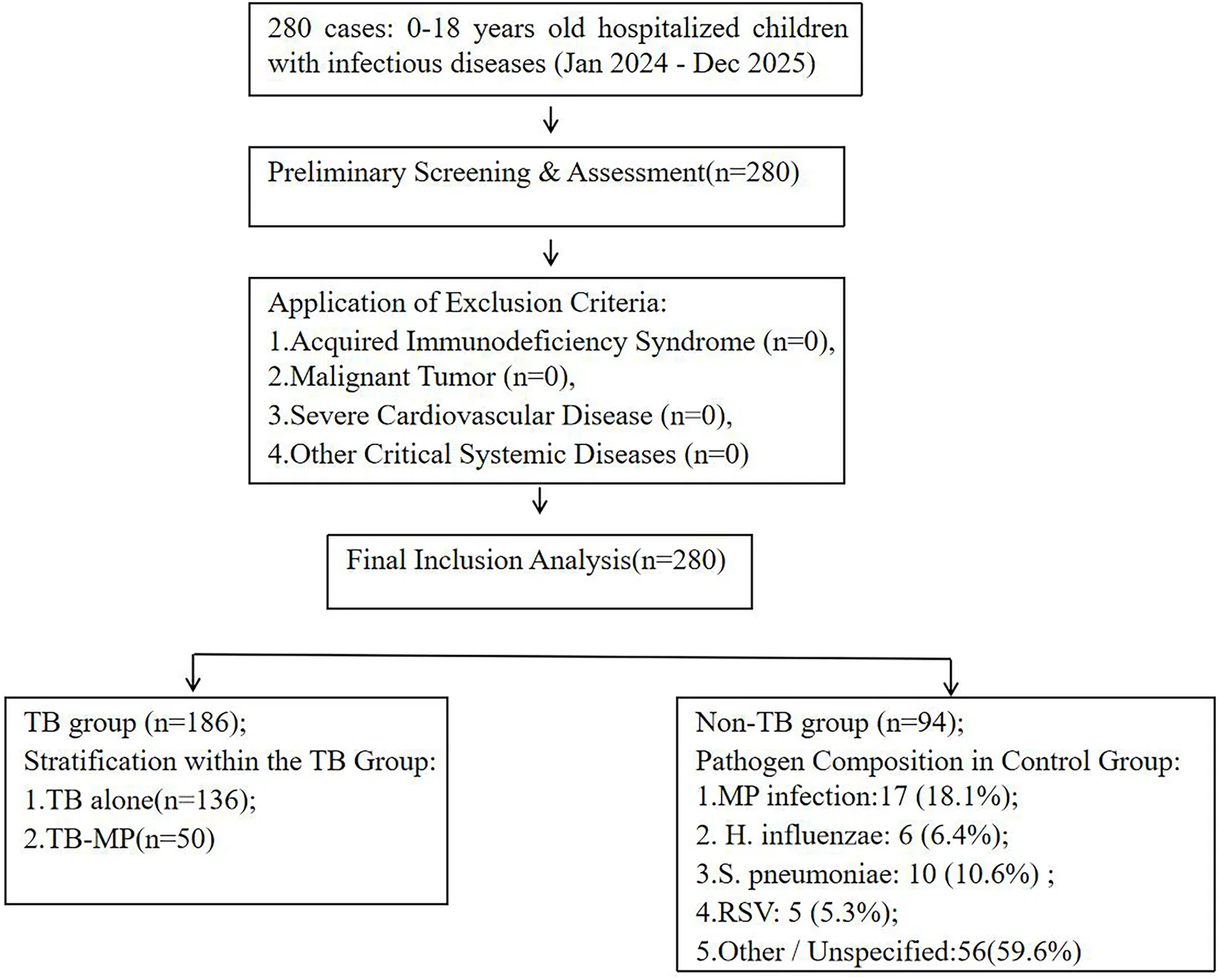
Flow diagram of patient selection. A total of 280 children aged 0–18 years with infectious diseases were screened between January 2024 and December 2025. After applying exclusion criteria (acquired immunodeficiency syndrome, malignant tumors, severe cardiovascular diseases, and other critical systemic disorders), all 280 patients were enrolled and classified into the TB group (n=186) and the non-TB control group (n=94) according to WHO diagnostic criteria. Within the TB group, 136 patients had isolated TB infection and 50 had TB-MP co-infection. The non-TB control group comprised MP infection (n=17, 18.1%), H. influenzae (n=6, 6.4%), S. pneumoniae (n=10, 10.6%), RSV (n=5, 5.3%), and other or unspecified pathogens (n=56, 59.6%). TB, tuberculosis; MP, Mycoplasma pneumoniae; RSV, respiratory syncytial virus.

### 3.2 Baseline characteristics and infection profiles of the study population

A total of 280 children were enrolled in this study, including 186 patients in the TB group and 94 subjects in the non-TB control group. As presented in **Table 1**, the proportion of male patients was marginally lower in the TB group than that in the non-TB group (49.4% vs. 58.5%), whereas vitamin D deficiency was more prevalent among children with TB (36.0% vs. 16.0%). MP co-infection was identified in 26.9% of the TB group and 18.1% of the non-TB group. With regard to other respiratory pathogens, infections caused by Haemophilus influenzae, Streptococcus pneumoniae and respiratory syncytial virus (RSV) were more frequent in the non-TB group (6.4%, 10.6% and 5.3%, respectively) relative to the TB group (1.0% for both Haemophilus influenzae, Streptococcus pneumoniae). Within the TB cohort, microbiological confirmation of tuberculosis was obtained in 5.9% of patients via acid-fast bacilli staining, 10.8% via mycobacterial culture, and 26.9% via TB-DNA nucleic acid testing. In addition, the median age was significantly higher in the TB group than in the non-TB control group (13.62 vs. 8.34 years, P < 0.001) (**Tables 1**, **2**), indicating a marked baseline age imbalance that was addressed through age adjustment, stratification, interaction testing, and age-matched sensitivity analyses in subsequent analyses.

**Table 1:** Baseline characteristics and infection profiles of the study population.

Parameter	TB group (n=186)	Non-TB control group (n=94)
Male sex [n (%)],	92 (49.4)	55 (58.5)
Age (years), median [IQR]	13.62 [11.33, 15.92]	8.34 [3.94, 14.37]
Vitamin D deficiency [n (%)]	67 (36.0)	15 (16.0)
MP infection [n (%)]	50 (26.9)	17 (18.1)
H. influenzae infection [n (%)]	2 (1.0)	6 (6.4)
S. pneumoniae infection [n (%)]	2 (1.0)	10 (10.6)
respiratory syncytial virus [n (%)]*	0 (0.0)	5 (5.3)
Acid-fast bacilli positive [n (%)]	11 (5.9)	—
Mycobacterial culture positive [n (%)]	20 (10.8)	—
TB-DNA positive [n (%)]	50 (26.9)	—

Data are presented as n (%). MP, Mycoplasma pneumoniae; TB, tuberculosis. “—” indicates not applicable.; *RSV cases in the control group (n=5) were excluded from the MP-only sensitivity analysis due to the absence of RSV in the TB group;.

**Table 2:** Comparison of immune-inflammatory parameters between the non-TB control group and the TB group.

Parameter	Control group (n=94), median [IQR]	TB group (n=186), median [IQR]	Z-value	P-value
Age (years)	8.34 [3.94, 14.37]	13.62 [11.33, 15.92]	−5.092	<0.001
CD4+ T (cells/μL)	767.00 [575.25, 1322.25]	639.50 [490.75, 824.25]	−3.360	0.001
CD8+ T (cells/μL)	567.50 [394.50, 758.50]	401.50 [319.00, 521.25]	−5.257	<0.001
NK cells (cells/μL)	234.50 [126.75, 373.75]	133.00 [87.00, 220.00]	−5.067	<0.001
B lymphocytes (cells/μL)	360.00 [224.00, 617.75]	284.00 [206.50, 385.25]	−2.836	0.005
PLT (×10^9^/L)	296.50 [214.25, 392.50]	277.50 [234.00, 325.75]	−1.314	0.189
NEUT (×10^9^/L)	3.77 [2.75, 6.15]	3.47 [2.56, 4.67]	−1.891	0.059
LYM (×10^9^/L)	2.48 [1.87, 3.31]	1.80 [1.44, 2.34]	−5.260	<0.001
MONO (×10^9^/L)	0.50 [0.32, 0.72]	0.41 [0.31, 0.56]	−2.769	0.006
NLR	1.65 [0.87, 2.32]	1.90 [1.20, 3.02]	−1.986	0.047
SII	413.39 [266.55, 735.19]	516.37 [307.99, 859.86]	−1.672	0.086
MLR	0.19 [0.14, 0.28]	0.22 [0.15, 0.31]	−1.287	0.149
PLR	117.63 [75.34, 163.08]	145.69 [114.25, 193.25]	−4.418	<0.001
SIRI	0.64 [0.37, 1.56]	0.75 [0.43, 1.32]	−0.033	0.905

Data are presented as median [interquartile range]. P-values were calculated using the Mann–Whitney U test. P < 0.05 was considered statistically significant. NLR, neutrophil-to-lymphocyte ratio; SII, systemic immune-inflammation index; MLR, monocyte-to-lymphocyte ratio; PLR, platelet-to-lymphocyte ratio; SIRI, systemic inflammation response index.

### 3.3 Comparison of immune-inflammatory parameters between the TB group and non-TB control group

We further compared immune-inflammatory parameters between the TB group (n = 186) and the non-TB control group (n = 94) using the Mann-Whitney U test (**Table 2**). The TB group had a significantly higher median age than the control group (13.62 vs. 8.34 years, P < 0.001). For lymphocyte subsets, the TB group presented markedly lower counts of CD4^+^ T cells (P = 0.001), CD8^+^ T cells (P < 0.001), natural killer (NK) cells (P < 0.001) and B lymphocytes (P = 0.005) relative to controls.

As for routine hematological indicators, the TB group showed significantly reduced lymphocyte (LYM, P < 0.001) and monocyte (MONO, P = 0.006) counts. Neutrophil (NEUT) counts tended to be slightly lower in the TB group, with a borderline statistical difference (P = 0.059). Platelet (PLT) counts were comparable across the two groups (P = 0.189). Among combined inflammatory indexes, the platelet-to-lymphocyte ratio (PLR) was significantly elevated in the TB group (P < 0.001), accompanied by a significant increase in the neutrophil-to-lymphocyte ratio (NLR, P = 0.047). In contrast, SII (P = 0.086), MLR (P = 0.149) and SIRI (P = 0.905) showed no statistically significant intergroup differences.

### 3.4 Univariate and multivariate logistic regression analyses of factors associated with pediatric tuberculosis

To screen independent influencing factors of pediatric tuberculosis, univariate and multivariate logistic regression analyses were conducted (**Table 3**). Univariate analysis showed that age, CD4^+^ T cells, CD8^+^ T cells, NK cells, B lymphocytes, white blood cell count (WBC), neutrophil count (NEUT), monocyte count (MONO), lymphocyte count (LYM) and vitamin D deficiency were significantly correlated with pediatric TB (all P < 0.05).

**Table 3:** Univariate and multivariate logistic regression analyses of factors associated with pediatric tuberculosis.

Variable	Unadjusted OR (95% CI)	P-value (unadjusted)	Adjusted OR (95% CI)	P-value (adjusted)
Gender	1.441 (0.873, 2.378)	0.153	1.106 (0.641, 1.908)	0.718
Age (per 1-year increase)	1.156 (1.098, 1.217)	<0.001	1.118 (1.046, 1.194)	0.001
LYM	0.617 (0.497, 0.766)	<0.001	0.814 (0.632, 1.050)	0.113
MP co-infection	1.665 (0.898, 3.087)	0.105	0.567 (0.292, 1.099)	0.093
CD4+ T	0.999 (0.998, 0.999)	<0.001	— (not included)‡	—
CD4+ T/CD8+ T	0.899 (0.683, 1.183)	0.445	— (not included)‡	—
CD8+ T	0.998 (0.997, 0.999)	<0.001	— (not included)‡	—
NK	0.996 (0.994, 0.998)	0.001	— (not included)‡	—
B lymphocytes	0.999 (0.998, 0.999)	0.001	— (not included)‡	—
WBC	0.849 (0.785, 0.919)	<0.001	— (not included)‡	—
PLT	0.998 (0.995, 1.000)	0.193	— (not included)‡	—
NEUT	0.908 (0.836, 0.986)	0.021	— (not included)‡	—
MONO	0.265 (0.118, 0.598)	0.001	— (not included)‡	—
Vitamin D deficiency (Yes vs. No)	2.965 (1.582, 5.556)	0.001	— (not included)§	—
Model Diagnostics¶	Hosmer-Lemeshow χ^2^ (df=8)	—	2.333, P = 0.969	—
	Nagelkerke R^2^	—	0.182	—

^†^Multivariate model was adjusted for age, sex, total lymphocyte count (LYM), and Mycoplasma pneumoniae (MP) co-infection status.

^‡^Lymphocyte subsets (CD4+ T and B cells) exhibited strong positive correlation with LYM (Pearson’s r > 0.6), while CD8+ T and NK cells showed moderate correlation with LYM (Pearson’s r = 0.4–0.6). All lymphocyte subsets were excluded to eliminate multicollinearity. WBC, platelet, neutrophil, and monocyte counts were further excluded to reduce redundant inflammatory predictors and avoid model overfitting.

^§^Vitamin D deficiency was not included in the primary multivariate model because of limited positive events in the control group (n = 15), which would violate the events-per-variable (EPV) rule. Sensitivity analysis adjusting for age, sex, and vitamin D status (without LYM) showed that the adjusted OR of age remained stable (OR ≈ 1.143, P < 0.001).

^¶^Hosmer–Lemeshow test: χ^2^ = 2.333, df = 8, P = 0.969, indicating favorable model calibration. Nagelkerke R^2^ = 0.182.

The multivariate regression model was adjusted for gender, age, LYM and MP co-infection status. The results revealed that age was the sole associated variable significantly associated with pediatric TB (adjusted OR = 1.118, 95% CI: 1.046–1.194, P = 0.001). After adjusting for age, lymphocyte count lost statistical significance (adjusted OR = 0.814, 95% CI: 0.632–1.050, P = 0.113), as did MP co-infection (adjusted OR = 0.567, 95% CI: 0.292–1.099, P = 0.093); gender showed no significant correlation with TB (P = 0.718).

CD4^+^ T cells, CD8^+^ T cells, NK cells and other leukocyte subsets were excluded from the primary multivariate model owing to collinearity with LYM (**Supplementary Table S1**). Vitamin D deficiency was also eliminated to prevent model overfitting, given the limited number of positive cases in the control group (n = 15). Sensitivity analysis verified that the statistical effect of age remained stable after further adjustment for vitamin D status. The multivariate model exhibited favorable goodness-of-fit (Hosmer–Lemeshow test: χ^2^ = 2.333, df = 8, P = 0.969; Nagelkerke R^2^ = 0.182), suggesting no obvious deviation between model predicted values and actual observed outcomes.

In sensitivity analyses, we substituted LYM with CD4^+^ T-cell count or vitamin D deficiency in the multivariate model. The adjusted OR for age remained essentially unchanged in both alternative models (CD4^+^ T model: OR ≈ 1.124, P < 0.001; vitamin D model: OR ≈ 1.143, P < 0.001). Notably, although vitamin D deficiency emerged as a significant independent correlate of pediatric TB (adjusted OR = 2.096, 95% CI: 1.083–4.057, P = 0.028), its inclusion did not attenuate the age effect (OR changed from 1.118 to 1.143). These results confirm that the independent predictive effect of age is robust and unaffected by the choice of immune-related or nutritional marker incorporated into the model (**Supplementary Table S2**).

### 3.5 Impact of *Mycoplasma pneumoniae* co-infection on immune-inflammatory profiles within the TB cohort

To evaluate whether Mycoplasma pneumoniae (MP) co-infection is associated with immune suppression in children with tuberculosis (TB) compared with isolated TB infection, we performed analysis of covariance (ANCOVA) adjusted for age to compare immune-inflammatory parameters between children with isolated TB (n = 136) and those with TB-MP co-infection (n = 50) (**Table 4**).

**Table 4:** Age-adjusted comparison of immune-inflammatory parameters between TB alone and TB-MP co-infection groups within the TB cohort*.

Indicator†	TB alone (n=136)	TB-MP co-infection (n=50)	Adjusted mean difference (95% CI)‡	P-value§	Partial η^2^
Lymphocyte Subsets					
CD4+ T (cells/μL)	639.50 (487.50–822.25)	637.00 (497.75–847.50)	0.020 (−0.109 to 0.149)	0.750	0.001
CD8+ T (cells/μL)	394.00 (305.25–514.25)	402.50 (355.75–541.25)	−0.057 (−0.192 to 0.079)	0.413	0.004
NK cells (cells/μL)	128.00 (85.25–212.00)	156.00 (91.25–252.50)	−0.120 (−0.321 to 0.081)	0.240	0.008
B lymphocytes (cells/μL)	277.50 (210.50–386.00)	290.50 (195.00–371.75)	0.056 (−0.114 to 0.215)	0.491	0.003
Routine Blood Parameters					
LYM (×10^9^/L)	1.80 (1.43–2.32)	1.75 (1.45–2.47)	0.021 (−0.091 to 0.132)	0.713	0.001
NEUT (×10^9^/L)	3.55 (2.51–4.69)	3.35 (2.59–4.48)	0.036 (−0.138 to 0.209)	0.687	0.001
MONO (×10^9^/L)	0.42 (0.32–0.58)	0.40 (0.29–0.51)	0.094 (−0.050 to 0.237)	0.198	0.009
PLT (×10^9^/L)	271.50 (233.25–327.25)	280.00 (239.00–324.00)	0.010 (−0.084 to 0.104)	0.832	0.000
Inflammatory Indices¶					
ln (NLR)	0.607	0.591	0.016 (−0.201 to 0.233)	0.887	0.000
ln (PLR)	4.987	4.997	0.011 (−0.154 to 0.132)	0.883	0.000
ln (SII)	6.236	6.212	0.025 (−0.239 to 0.289)	0.852	0.000
ln (SIRI)	−0.224	−0.331	0.107 (−0.202 to 0.416)	0.496	0.003

*All data were analyzed using general linear models (ANCOVA) with age as a covariate.

^†^Data for lymphocyte subsets and routine blood parameters are presented as median (interquartile range). For log-transformed inflammatory indices, geometric means (back-transformed from the log scale) are presented.

^‡^Mean difference = (TB-MP co-infection group) − (TB alone group). Adjusted mean differences are presented on the natural log-transformed scale for lymphocyte subsets and inflammatory indices.

^§^P < 0.05 was considered statistically significant after adjustment for age.

^¶^Due to skewed distributions, NLR, PLR, SII, SIRI, and MLR were natural log-transformed prior to analysis.

After adjustment for age, no statistically significant intergroup differences were identified for lymphocyte subsets (CD4^+^ T cells, CD8^+^ T cells, NK cells and B lymphocytes) or routine hematological indicators (LYM, NEUT, MONO and PLT), with all P values > 0.05. The partial η^2^ of all parameters was less than 0.01, demonstrating trivial effect sizes and no clinically relevant intergroup trends.

All composite inflammatory indices (NLR, PLR, SII and SIRI) exhibited skewed distributions and were therefore log-transformed before statistical testing; still, no significant disparities were found between the two groups (all P > 0.05), with partial η^2^ ranging from 0.000 to 0.003, reflecting nearly negligible group effects. To further evaluate whether MP co‐infection has an independent effect on immune parameters, we conducted a descriptive sensitivity analysis comparing MP‐only controls with TB‐MP co‐infected cases. Descriptively, MP‐only controls had higher CD8^+^ T cell counts [583.50 (335.25–788.25) vs. 402.50 (355.75–541.25) cells/μL] and lymphocyte counts [2.67 (1.94–3.57) vs. 1.75 (1.45–2.47) ×10^9^/L] compared with TB‐MP co‐infected patients, suggesting that MP infection alone does not appear to produce the degree of lymphocyte reduction observed in TB patients (**Supplementary Table S3**).

### 3.6 Diagnostic performance of age and age-combined models

The single-variable model of age produced an AUC of 0.686 (95% CI: 0.618–0.755). For the models combining age with NLR, SII, PLR, or MP co-infection status, the AUC values were 0.694 (95% CI: 0.626–0.762), 0.690 (95% CI: 0.622–0.759), 0.711 (95% CI: 0.644–0.778), and 0.705 (95% CI: 0.639–0.770), respectively (**Figure 2**), with AUC differences ranging from 0.004 to 0.025 compared with age alone; none reached statistical significance (P = 0.494, 0.662, 0.166, and 0.075, respectively). The model combining age with CD8^+^ T cell count yielded the highest AUC of 0.727 (95% CI: 0.664–0.790), with an AUC difference of 0.041 (DeLong test P = 0.009). Although this AUC improvement was statistically significant, its clinical utility—as further evaluated by NRI, IDI, and decision curve analyses—was modest and primarily limited to intermediate threshold ranges (**Supplementary Table S4**; **Supplementary Figure S2**). All pairwise comparisons for the age-combined models are detailed in **Table 5** and **Supplementary Table S5**.

**Figure 2:**
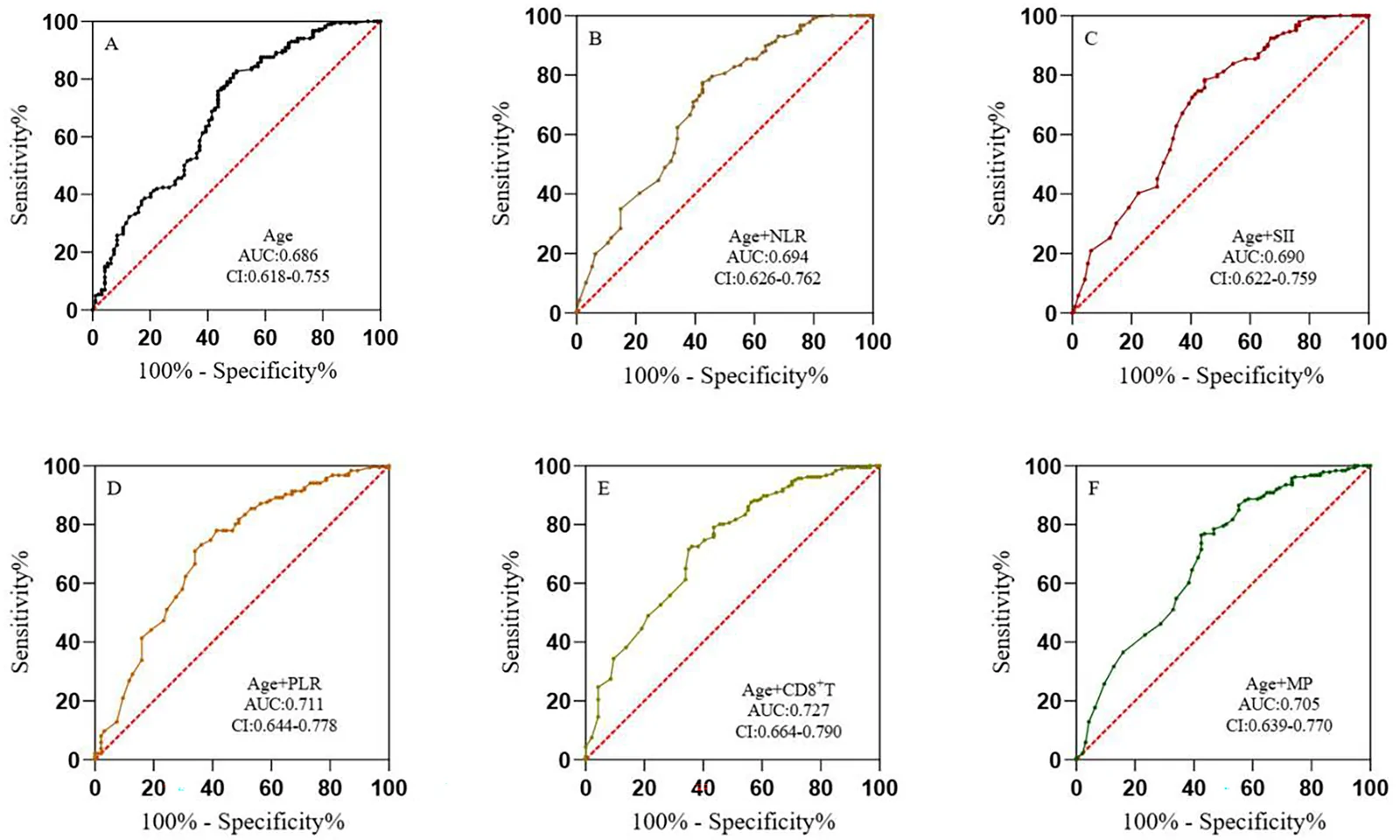
Diagnostic performance of the age-only model and age-combined predictive models for pediatric tuberculosis. ROC curves comparing the age-only model with models combining age and various peripheral blood biomarkers (NLR, SII, PLR, MP infection status, and CD8^+^ T cell count). The diagonal dashed line represents the reference line of no discrimination (AUC = 0.5). AUC values with 95% confidence intervals (CIs) for each model are presented in **Table 5**. Black line: age-only model; Orange line: age + NLR model; Red line: age + SII model; Dark yellow line: age + PLR model; Light green line: age + CD8^+^T model; Dark green line: age + MP model; Abbreviations: AUC, area under the curve; CI, confidence interval; MP, Mycoplasma pneumoniae; NLR, neutrophil-to-lymphocyte ratio; PLR, platelet-to-lymphocyte ratio; ROC, receiver operating characteristic; SII, systemic immune-inflammation index.

**Table 5:** ROC curve comparisons between the age-only model and age-combined predictive models.

Model comparison	AUC (combined model)	AUC (age alone)	AUC difference† (95% CI)	DeLong Z	P-value
Age + NLR vs. Age	0.694	0.686	0.008 (-0.015~0.031)	0.683	0.494
Age + SII vs. Age	0.690	0.686	0.004 (-0.014~0.023)	0.437	0.662
Age + PLR vs. Age	0.711	0.686	0.025 (-0.010~0.059)	1.387	0.166
Age + MP vs. Age	0.705	0.686	0.018 (-0.002~0.039)	1.783	0.075
Age + CD8+ T vs. Age	0.727	0.686	0.041 (0.010~0.071)	2.615	0.009

†AUC difference = AUC(combined model) − AUC(age alone). Positive values indicate superior performance of the combined model. 95% CIs for AUC differences were eAbbreviations: AUC, area under the curve; CI, confidence interval; MP, Mycoplasma pneumoniae; NLR, neutrophil-to-lymphocyte ratio; PLR, platelet-to-lymphocyte ratio; SII, systemic immune-inflammation index; TB, tuberculosis.

### 3.7 Comprehensive evaluation of model performance

Beyond discrimination, we assessed calibration and clinical utility [as shown in **Supplementary Figure S1** (calibration curves) and S2 (decision curve analysis), **Supplementary Table S4**]. The Age + CD8^+^ T-cell model demonstrated good calibration (Hosmer–Lemeshow χ^2^ = 10.57, P = 0.228; Brier score = 0.187; McFadden R^2^ = 0.127), which was comparable to or slightly better than the Age-only model (HL χ^2^ = 14.61, P = 0.067; Brier score = 0.195; McFadden R^2^ = 0.094; relative improvement 35%). Decision curve analysis showed that, across clinically relevant threshold probabilities (approximately 0.40 to 0.78), the Age + CD8^+^ T-cell model consistently provided higher net benefit than the Age-only model. Quantitative reclassification metrics further supported the incremental value of CD8^+^ T-cell counts: continuous NRI = 0.505 (95% CI 0.258–0.755; P < 0.001), event NRI^+^ = 0.419, non-event NRI^-^ = 0.085, and IDI = 0.038 (95% CI 0.011–0.068; P = 0.003). These findings indicate that the addition of CD8^+^ T-cell counts improves risk prediction not only in ranking accuracy but also in clinical decision-making.

### 3.8 Age-stratified comparison of immune-inflammatory indicators between TB and non-TB groups

To explore age-dependent heterogeneity in immune-inflammatory parameters, participants were divided into three age strata (<5 years, 5–12 years, and ≥12 years), and immune-inflammatory indicators were compared between TB and non-TB groups within each stratum (**Table 6**).

**Table 6:** Comparison of immune-inflammatory indicators between TB and non-TB children across three age strata §.

Indicator	TB group, median (IQR)	Non-TB group, median (IQR)	Z	P	Adjusted P-value*†*	r*‡*
<5 years (TB: n=14, Non-TB: n=30)¶						
CD4+ T (cells/μL)	1182.00 (842.00–2162.75)	1559.50 (877.00–2519.75)	−0.857	0.392	1.000	0.129
CD8+ T (cells/μL)	709.50 (452.50–828.25)	782.50 (459.00–918.50)	−0.391	0.696	1.000	0.059
NK cells (cells/μL)	288.00 (183.00–343.25)	318.00 (185.50–514.75)	−0.995	0.320	1.000	0.150
B cells (cells/μL)	905.50 (575.50–1336.75)	924.00 (454.50–1520.00)	0.000	1.000	1.000	0
NEUT (×10^9^/L)	2.31 (1.74–3.60)	3.52 (2.19–6.46)	−1.890	0.059	0.472	0.285
LYM (×10^9^/L)	3.80 (3.06–5.55)	4.14 (3.09–5.54)	−0.050	0.960	1.000	0.008
NLR	0.67 (0.32–0.94)	0.84 (0.60–1.28)	−1.512	0.131	0.917	0.228
SII	170.00 (133.65–341.92)	330.51 (236.29–486.13)	−2.091	0.036	0.324	0.315
PLR	82.45 (66.42–99.66)	93.16 (68.70–142.81)	−1.285	0.199	1.000	0.193
5–12 years (TB: n=41, Non-TB: n=23)						
CD4+ T (cells/μL)	665.00 (501.50–823.50)	732.00 (494.00–1014.00)	−0.672	0.502	1.000	0.084
CD8+ T (cells/μL)	404.00 (321.00–551.50)	506.00 (371.00–694.00)	−1.826	0.068	0.544	0.228
NK cells (cells/μL)	162.00 (93.00–304.00)	213.00 (126.00–379.00)	−0.623	0.533	1.000	0.078
B cells (cells/μL)	287.00 (248.50–430.50)	280.00 (184.00–425.00)	−0.581	0.561	1.000	0.073
NEUT (×10^9^/L)	3.40 (2.21–4.71)	3.34 (2.61–5.96)	−0.679	0.497	1.000	0.084
LYM (×10^9^/L)	1.87 (1.42–2.79)	2.44 (1.77–2.85)	−1.609	0.108	0.756	0.201
NLR	1.69 (1.18–2.34)	1.54 (1.06–2.06)	−0.266	0.790	1.000	0.033
SII	519.15 (288.06–926.59)	415.46 (257.36–711.40)	−0.735	0.463	1.000	0.092
PLR	158.72 (116.49–197.10)	121.66 (87.72–163.40)	−2.330	0.020	0.180	0.291
≥12 years (TB: n=131, Non-TB: n=41)						
CD4+ T (cells/μL)	594.00 (471.00–770.00)	640.00 (506.00–788.50)	−0.740	0.459	1.000	0.056
CD8+ T (cells/μL)	382.00 (300.00–500.00)	542.00 (394.00–634.00)	−3.996	<0.001	<0.009	0.305
NK cells (cells/μL)	121.00 (82.00–171.00)	187.00 (115.50–290.00)	−3.527	<0.001	<0.008	0.269
B cells (cells/μL)	262.00 (195.00–345.00)	285.00 (165.50–396.00)	−0.518	0.605	1.000	0.040
NEUT (×10^9^/L)	3.63 (2.65–4.68)	4.05 (3.27–5.98)	−2.115	0.034	0.170	0.161
LYM (×10^9^/L)	1.70 (1.41–2.12)	2.01 (1.59–2.53)	−2.485	0.013	0.078	0.190
NLR	2.06 (1.47–3.59)	2.22 (1.54–2.99)	−0.487	0.626	1.000	0.037
SII	560.30 (345.34–963.88)	530.27 (276.29–894.68)	−0.613	0.540	1.000	0.047
PLR	150.33 (122.94–201.99)	124.12 (76.35–181.14)	−2.641	0.008	0.056	0.201

^†^P-values were calculated using the Mann–Whitney U test and adjusted for multiple comparisons using the Holm correction independently within each age stratum. Adjusted P-values are presented alongside the original values. Holm-adjusted P-values > 1.000 were truncated to 1.000.

^‡^Effect sizes (r) were calculated as r = Z/√N and interpreted as small (≥0.1), medium (≥0.3), or large (≥0.5). Due to the limited sample sizes in certain subgroups, 95% confidence intervals for effect sizes were not calculated.

^§^Given the exploratory nature of these analyses, all subgroup results should be interpreted with caution and considered hypothesis-generating.

^¶^Sensitivity power analysis indicated that the minimum detectable effect size for the <5 years stratum (TB: n=14, control: n=30; α=0.05, power=0.80) was Cohen’s d = 0.93, corresponding to a large effect.

In the <5 years stratum, no statistically significant differences were observed between TB and non-TB groups for any of the tested indicators after Holm correction (all adjusted P-values > 0.05). Effect sizes (r) ranged from 0.000 to 0.315, with NEUT (r = 0.285) and SII (r = 0.315) approaching medium effects, but none reached statistical significance. Sensitivity power analysis showed that the minimum detectable effect size was Cohen’s d = 0.93, corresponding to a large effect, based on the available sample sizes (TB: n=14, control: n=30).

In the 5–12 years stratum, no statistically significant differences were observed for any of the tested indicators after Holm correction (all adjusted P-values > 0.05). Effect sizes ranged from 0.033 to 0.291, with PLR demonstrating the largest effect (r = 0.291), approaching the medium threshold.

In the ≥12 years stratum, CD8^+^ T cells (median: 382.00 vs. 542.00 cells/μL) and NK cells (median: 121.00 vs. 187.00 cells/μL) remained significantly lower in the TB group after Holm correction (adjusted P < 0.009 and P < 0.008, respectively), with medium (r = 0.305) and approaching-medium (r = 0.269) effect sizes. In contrast, PLR, LYM, and NEUT lost significance after correction (adjusted P = 0.056, 0.078, and 0.170, respectively), and no significant differences were observed for CD4^+^ T cells, B cells, NLR, or SII (all adjusted P-values = 1.000).

Collectively, these findings indicate that TB-associated immune-inflammatory differences were predominantly concentrated in the ≥12 years stratum, particularly for CD8^+^ T cells and NK cells, whereas no significant differences were detected in the <5 years or 5–12 years strata after correction for multiple comparisons. Given the exploratory nature of these age-stratified analyses, all subgroup results should be interpreted with caution and considered hypothesis-generating.

### 3.9 Validation of age-dependent immune heterogeneity by interaction analyses

To formally test whether age modifies the effect of TB on immune-inflammatory parameters, we performed Age × Group interaction analyses (**Table 7**). The results revealed significant interactions for CD8^+^ T cells (P < 0.001, partial η^2^ = 0.089), NK cells (P < 0.001, partial η^2^ = 0.196), LYM (P < 0.001, partial η^2^ = 0.404), and PLR (P < 0.001, partial η^2^ = 0.088), confirming that age is a significant effect modifier for these TB-associated immune alterations. LYM showed the largest effect size (partial η^2^ = 0.404), followed by B lymphocytes (partial η^2^ = 0.359) and CD4^+^ T cells (partial η^2^ = 0.344). Additionally, MONO (partial η^2^ = 0.102) and NLR (partial η^2^ = 0.081) also showed significant interactions (both P < 0.001). In contrast, NEUT (P = 0.034, partial η^2^ = 0.024) and SII (P = 0.025, partial η^2^ = 0.026) showed marginal interactions, whereas SIRI showed no significant interaction (P = 0.281, Partial η^2^ = 0.009). These interaction results are fully consistent with the age-dependent immune heterogeneity observed in the age-stratified comparisons (**Table 6**), further supporting age as a core stratification factor in pediatric TB immune assessment.

**Table 7:** Age × Group interaction effects on immune-inflammatory parameters.

Immune parameter	F-value	df	P for interaction	Partial η^2^
CD4^+^ T cells (cells/μL)	72.675	2	<0.001	0.344
CD8^+^ T cells (cells/μL)	13.566	2	<0.001	0.089
NK cells (cells/μL)	33.660	2	<0.001	0.196
B lymphocytes (cells/μL)	77.730	2	<0.001	0.359
LYM (×10^9^/L)	93.826	2	<0.001	0.404
NEUT (×10^9^/L)	3.414	2	0.034	0.024
MONO (×10^9^/L)	15.699	2	<0.001	0.102
NLR	12.232	2	<0.001	0.081
SII	3.724	2	0.025	0.026
PLR	13.364	2	<0.001	0.088
SIRI	1.274	2	0.281	0.009

df, degrees of freedom; LYM, lymphocyte; MONO, monocyte; NEUT, neutrophil; NLR, neutrophil-to-lymphocyte ratio; PLR, platelet-to-lymphocyte ratio; SII, systemic immune-inflammation index; SIRI, systemic inflammation response index.

Data were analyzed using univariate general linear models with age as a continuous covariate and Group (TB vs. non-TB) as a fixed factor. P for interaction < 0.05 indicates significant effect modification by age. Effect sizes were expressed as partial η^2^, interpreted according to Cohen’s conventions (small ≥ 0.01, medium ≥ 0.06, large ≥ 0.14).

### 3.10 Age-matched sensitivity analysis

To exclude age confounding, we performed a 1:1 age-matched sensitivity analysis. A total of 83 matched pairs (83 TB patients and 83 age-matched controls) were successfully generated, with well-balanced age between groups after matching (P = 0.718). Immune parameters were compared between matched pairs using the Wilcoxon signed-rank test, and the analysis was repeated in the ≥12 years stratum (41 pairs) (**Supplementary Table S6**).

In the overall matched cohort, CD8^+^ T cells (TB vs. control: 441.0 vs. 548.0 cells/μL, P < 0.001), NK cells (138.0 vs. 213.0 cells/μL, P = 0.005), lymphocytes (2.05 vs. 2.43 ×10^9^/L, P = 0.017), and PLR (137.2 vs. 116.8, P = 0.005) remained significantly different between TB patients and age-matched controls. In contrast, CD4^+^ T cells lost significance after matching (665.0 vs. 732.0 cells/μL, P = 0.113), supporting the reviewer’s interpretation that this parameter is primarily age-driven. B cells (P = 0.663) and monocytes (P = 0.573) also showed no significant differences after matching.

In the ≥12 years stratum, CD8^+^ T cells (P = 0.004) and NK cells (P < 0.001) remained highly significant, with effect sizes even more pronounced than in the overall matched cohort. This further corroborates our primary findings (**Table 6**) and interaction analysis (**Table 7**)—the reductions in CD8^+^ T cells and NK cells in adolescents are associated with TB status rather than age confounding. Lymphocytes (P = 0.024) and PLR (P = 0.034) also remained significant in this stratum, whereas CD4^+^ T cells (P = 0.569), B cells (P = 0.385), and monocytes (P = 0.985) did not.

WBC showed a significant difference in the overall matched cohort (P = 0.013) but lost significance in the ≥12 years stratum (P = 0.315), suggesting that this difference may be partially driven by age or other confounding factors in younger children.

### 3.11 Comparison of immune parameters between bacteriologically confirmed and clinically diagnosed TB cases

To further evaluate the potential impact of misclassification bias, we compared immune parameters between bacteriologically confirmed (n=68) and clinically diagnosed (n=118) TB cases using ANCOVA with age as a covariate (**Supplementary Table S7**). After age adjustment, no statistically significant differences were observed between the two subgroups for any of the tested parameters (all P > 0.05), with all Partial η^2^ values ≤ 0.017, indicating negligible to small effect sizes. These findings suggest that clinically diagnosed cases were immunologically consistent with confirmed cases, and that misclassification bias is unlikely to have materially affected our primary conclusions.

## 4 Discussion

The diagnosis of pediatric tuberculosis relies on an integrated evidence chain comprising epidemiological exposure history, immunological assays (e.g., IGRAs, tuberculin skin test), and characteristic radiological manifestations—most notably hilar and mediastinal lymphadenopathy—rather than direct microbiological confirmation alone (10–15). This diagnostic complexity is further compounded by the age-dependent maturation of the immune system. In this real-world retrospective cohort study, we identified age as the sole independent correlate of pediatric TB and demonstrated marked age-dependent heterogeneity in immune-inflammatory profiles.

This study identified age as the sole associated variable correlated with pediatric tuberculosis, consistent with age-dependent immune maturation trajectories documented in prior research (16–18). Our results demonstrated that children with TB were significantly older than non-TB controls, accompanied by reduced counts of CD8^+^ T cells, NK cells, B lymphocytes, and total lymphocytes, as well as elevated PLR. Notably, CD4^+^ T cell counts displayed intergroup differences in the overall cohort comparison but not after age stratification, indicating that the initially observed CD4^+^ T cell decline may be largely driven by physiological age-related changes rather than tuberculous infection per se, although this interpretation remains associative rather than causal given the observational study design. This finding underscores the importance of distinguishing age-associated physiological immune shifts from TB-specific immunological alterations and suggests that CD4^+^ T cell counts may be particularly susceptible to confounding by age in pediatric populations. Mechanistically, childhood CD8^+^ T cell counts exhibit age-dependent dynamic changes (19), and mycobacterial infection may disrupt this trajectory; however, such disruption is masked in younger children by the substantial physiological fluctuations that characterize early immune development.

ROC curve analyses revealed that the diagnostic performance of the age-only model (AUC = 0.686) and all age-combined biomarker models remained moderate. Among all combined models, only the model integrating age with CD8^+^ T cell count showed a statistically significant improvement over the age-only model (AUC difference = 0.041; DeLong test P = 0.009; likelihood-ratio test P < 0.001). To further evaluate the clinical significance of this improvement, we performed NRI, IDI, and decision curve analyses (**Table 5**;**Supplementary Table S6**; **Supplementary Figure S2**). The category-free NRI was 0.505 (95% CI: 0.258–0.755, P < 0.001) and the IDI was 0.038 (95% CI: 0.011–0.068, P = 0.003), both reaching statistical significance. The event NRI^+^ was 0.419, indicating that approximately 41.9% of TB patients were correctly reclassified as high risk by the model. Decision curve analysis further demonstrated that the age + CD8^+^T model provided positive clinical net benefit across threshold probabilities of approximately 0.40 to 0.78. Collectively, although the AUC improvement was modest, the consistent evidence from multi-dimensional assessments suggests that the addition of CD8^+^T cell count may add incremental value in individual risk stratification and clinical decision-making.

Two predicted probability outliers (TB: 9.14%; non-TB: 3.75%) were confirmed to have error-free data, both attributable to markedly elevated CD8^+^T cell counts. Given the negative CD8 coefficient in the model, these high values substantially suppressed predictions. These two cases accounted for only 0.7% of the total sample and did not affect overall conclusions, but highlight the model’s sensitivity to extreme CD8^+^T cell values in certain clinical scenarios. Most critically, age-stratified analysis (**Table 6**) revealed that TB-associated immune differences were predominantly concentrated in the ≥12 years stratum, with CD8^+^ T cells and NK cells remaining significant after Holm correction, showing medium (r = 0.305) and approaching-medium (r = 0.269) effect sizes, respectively, whereas almost no detectable differences existed in children younger than 5 years. To formally test whether age modifies the effect of TB on immune parameters, we performed Age × Group interaction analyses, which confirmed significant effect modification by age for CD8^+^ T cells, NK cells, and lymphocyte count (**Table 7**), indicating that age modifies TB-related alterations in lymphocyte subsets. The convergence of these independent analytical approaches supports the notion that age should be regarded as a biological effect modifier rather than merely a demographic confounder in pediatric TB immune assessment. The age-matched sensitivity analysis further supported these conclusions. After matching for age, CD8^+^ T-cell and NK-cell differences remained significant, whereas CD4^+^ T-cell differences were abolished. This pattern confirms that the CD4^+^ T-cell difference is predominantly age-driven, while the CD8^+^ T-cell and NK-cell alterations in adolescents are TB-associated and independent of age.

This age-driven immunological heterogeneity directly explains why the overall AUC of the age-only model was only moderate: young children lack distinct TB-specific immune signatures, which dilutes the overall diagnostic signal when all age groups are pooled together. These findings suggest that circulating immunological biomarkers have limited diagnostic efficacy in early childhood, yet possess mild reference value for school-age children and adolescents. Furthermore, it should be emphasized that age is an intrinsic demographic attribute rather than a pathophysiological marker; the age-only AUC therefore reflects differences in case mix and healthcare-seeking patterns between the TB and non-TB populations rather than a causally informative diagnostic signal, and age cannot be regarded as a clinically deployable diagnostic test.

Beyond age-related heterogeneity, we also examined the impact of concurrent infections on systemic immune profiles. A novel and clinically meaningful finding of the present study from our intra-TB cohort analysis is that within the TB patient cohort, MP co-infection was not observed to induce remarkable alterations in immune-inflammatory parameters after adjustment for age (all P > 0.05, partial η^2^ < 0.01). We initially hypothesized that MP co-infection would exacerbate TB-related lymphopenia through synergistic inflammatory activation; however, our data do not provide strong support for this hypothesis within the limitations of our sample size. This finding may bears practical clinical implications: clinicians should neither presume that TB-suspected children with concurrent MP infection exhibit milder immune dysregulation nor rule out tuberculosis solely based on positive MP testing. Consistently low effect sizes (partial η^2^ < 0.01) further argue against the presence of synergistic immunosuppressive interactions between TB and MP in our study population, although larger studies would be required to definitively exclude small effect sizes. Modulatory effects of concurrent co-infections on systemic immune responses have been widely described in pediatric infectious disease research (20, 21). Nevertheless, our data suggest that once age is statistically controlled, MP co-infection exerts limited detectable influence on systemic immune-inflammatory indicators in children with TB in this cohort. This finding diverges from an adult-based study that reported synergistic inflammatory activation during co-infection, which may stem from age-related disparities in immune responsiveness (22). Children younger than 5 years and immunocompromised pediatric patients carry the highest risk of severe TB complications (7). For infants under 2 years old, IGRA testing is not prioritized due to insufficient diagnostic sensitivity, further underscoring the central role of age in diagnostic test selection (23).

A body of existing literature has validated the age dependency of cellular immune function (17, 18). Rothoeft et al. reported that IGRA sensitivity is markedly lower in children under 5 years compared with older pediatric and adult populations (17). Immature innate and adaptive immune systems in early life blunt the characteristic immune response triggered by Mycobacterium tuberculosis, which aligns with our observation that no TB-specific immunological differences could be identified in children younger than 5 years. Our real-world clinical data expand upon these prior findings, suggesting that age may be not merely a demographic confounding variable but a core determinant shaping immune-inflammatory phenotypes among pediatric TB patients. Transcriptomic research documenting progressive maturation of leukocyte subsets throughout childhood further corroborates this mechanistic interpretation (24).

Several studies have explored age-stratified TB immunity. Seddon et al. described the low TB risk observed in primary school-aged children (25). Dreesman et al. demonstrated that specific T-cell responses could distinguish active TB from latent TB infection in children, with different markers required for those under 3 years of age (26). A large cohort study further showed that age-dependent T lymphocyte subset reductions were predominantly observed in adults (27). The unique contributions of our study are fourfold. First, we used other common respiratory infections as controls, directly mirroring real-world clinical diagnostic scenarios. Second, we focused exclusively on children (0–18 years) with three age strata (<5, 5–12, ≥12 years) tailored to pediatric immune development. Third, we applied comprehensive model evaluation (AUC, calibration, DCA, NRI, and IDI) to quantify the incremental diagnostic value of biomarkers beyond age—an approach not previously used in this context. Fourth, we systematically assessed the impact of Mycoplasma pneumoniae co-infection on TB-related immune profiles after age adjustment. Collectively, our findings translate the established concept of age-dependent TB immunology into clinically actionable insights for pediatric TB diagnosis.

Overlapping clinical manifestations and radiological features between TB and MP infections frequently create diagnostic dilemmas in routine clinical practice (5, 28). The current study provides two key clinical clarifications: First, single routine inflammatory indices cannot reliably distinguish TB from MP-associated respiratory disease. Most composite inflammatory markers (SII, MLR, SIRI) showed no intergroup differences between TB and non-TB children, and ROC analyses confirmed that immune biomarkers yielded negligible incremental diagnostic value beyond age. Second, age stratification combined with multi-dimensional comprehensive evaluation—incorporating contact history, chest imaging and pathogen detection—remains indispensable for accurate differential diagnosis, especially for young children, in whom peripheral blood biomarkers provide the least diagnostic information.

Beyond the primary analyses of immune parameters, we also explored additional factors that may modulate immune-inflammatory profiles through sensitivity analyses (**Supplementary Table S2**). Vitamin D deficiency emerged as a significant independent correlate after adjusting for age (adjusted OR = 2.096, 95% CI: 1.083–4.057, P = 0.028). This finding is biologically plausible, given that vitamin D enhances the antimicrobial activity of macrophages against M. tuberculosis via the cathelicidin pathway (29). However, we emphasize that this association was derived from an exploratory sensitivity model with limited case numbers in the control group (n = 15) and should therefore be interpreted with caution—as hypothesis-generating rather than confirmatory. More importantly, adjusting for vitamin D status did not materially alter the adjusted OR for age (from 1.118 to 1.143), indicating that the dominant effect of age on TB susceptibility operates through pathways largely independent of vitamin D-mediated innate immunity or general nutritional status. Whether vitamin D deficiency represents a modifiable risk factor or merely a marker of increased TB susceptibility requires further prospective investigation.

From a translational clinical perspective, this real-world retrospective study proposes three practical clinical recommendations: (1) Age should be adopted as a core stratification indicator during pediatric TB assessment. Children aged <5 years display weak TB-specific immune signatures; clinicians should maintain high vigilance based on epidemiological exposure history, imaging manifestations and microbiological testing rather than over-reliance on peripheral blood immune biomarkers. (2) In our cohort, MP co-infection was not associated with aggravated TB-related lymphopenia. Tuberculosis cannot be excluded in MP-positive children with imaging findings consistent with TB, and clinicians should not judge the severity of immune dysfunction merely according to MP infection status based on the current evidence. (3) Peripheral blood lymphocyte biomarkers provide limited additional diagnostic value when combined with age. Although the age + CD8^+^ T cell model achieved statistically superior performance, its clinical utility was marginal. Therefore, routine lymphocyte subset detection may not be recommended as a universal screening tool for pediatric TB diagnosis based on our current findings, and could be considered in selective cases or research settings where immune phenotyping is of interest.

Several limitations of this single-center retrospective study deserve acknowledgment. First, inherent selection bias cannot be fully eliminated, and the retrospective design precludes causal inference. Second, the subgroup of children <5 years contained a relatively small number of TB cases (n = 14), limiting statistical power; sensitivity power analysis showed that this subgroup could only detect large effects (Cohen’s d ≥ 0.93). Third, the isolated MP infection subgroup included only 11 patients, precluding reliable subgroup comparison. Fourth, due to the retrospective design, MP nucleic acid testing was not performed in all patients, and MP-positive cases could not be reliably distinguished into PCR-confirmed versus serologically diagnosed categories, which may have introduced some degree of MP infection misclassification. However, the core conclusion of this study—that age is the key associated factor in pediatric TB—is primarily derived from the overall TB versus non-TB comparison rather than MP subgroup analyses; therefore, this limitation is unlikely to materially affect our main findings. Fifth, although formal correction for multiple testing (Holm correction) was applied in the age-stratified analyses, the relatively small sample sizes in certain subgroups limit the precision of these estimates; therefore, all subgroup results should be interpreted with caution and considered hypothesis-generating rather than definitive. Sixth, the age-matched analysis retained 83 TB patients (44.6% of the original TB cohort); the unmatched, older TB patients could not be included owing to the limited number of age-compatible controls. Seventh, the high proportion of clinically diagnosed TB cases (64.5%)—although consistent with the paucibacillary nature of childhood tuberculosis—may have introduced some misclassification bias; however, after age adjustment, no significant differences were observed between confirmed and clinically diagnosed cases for any immune parameter, suggesting that the impact of this bias on our primary conclusions is likely limited. Eighth, the predictive model evaluation was based on a case-control design (event rate 66.4%), which does not represent the general population prevalence of pediatric TB; therefore, extrapolation of predicted probabilities and decision curve analysis results to population-based screening settings should be interpreted with caution, and external validation in cohort-based studies is warranted. Finally, we did not assess serum albumin or anthropometric measures, which precludes distinguishing the independent contributions of vitamin D deficiency from those of generalized malnutrition or other micronutrient deficiencies. Future multi-center prospective longitudinal studies with serial immune biomarker monitoring are required to validate our age-stratified findings and characterize dynamic shifts in immune indicators during anti-tuberculosis treatment.

## 5 Conclusion

This real-world study identifies age as the key variable associated with pediatric tuberculosis and demonstrates marked age-dependent heterogeneity in immune-inflammatory profiles. After adjusting for age, MP co-infection was not associated with exacerbated TB-related lymphopenia in this cohort. Although the age + CD8^+^ T-cell model showed only a modest AUC improvement (0.041), the converging evidence from NRI (41.9% of TB patients correctly reclassified) and DCA (net benefit across threshold probabilities 0.40–0.78) supports the incremental value of CD8^+^ T-cell counts in individual risk stratification. Peripheral blood immune biomarkers added limited diagnostic information beyond age alone, and their clinical utility appears restricted to intermediate-risk scenarios rather than universal screening. These findings underscore that age stratification should be a core principle in pediatric TB evaluation and support the development of age-adapted diagnostic algorithms. For young children, clinical manifestations and imaging remain more reliable than blood immune biomarkers.

## Data Availability

The original contributions presented in the study are included in the article/**Supplementary Material**. Further inquiries can be directed to the corresponding author.

## Supplementary material

Supplementary Figure 1 Calibration plots for **(A)** the age-only model and **(B)** the age + CD8^+^ T-cell model. Patients were ranked by predicted probability and grouped into ten deciles of equal size (n = 28 per decile). The x-axis shows the mean predicted probability of tuberculosis in each decile; the y-axis shows the observed proportion of tuberculosis cases. The dashed diagonal line represents perfect calibration (predicted = observed). Hosmer–Lemeshow P values were 0.067 for Model A and 0.228 for Model B; Brier scores were 0.195 and 0.187, respectively. Blue line: Age-only model; Red line: Age+CD8^+^T-cell model.

Supplementary Figure 2 Decision curve analysis. Comparison of clinical net benefit between the age-only model and the age+CD8^+^T cell model across a range of threshold probabilities. The dashed gray line represents the “treat all” strategy; the solid black line represents the “treat none” strategy (net benefit = 0). The green line represents the age-only model, and the red line represents the age+CD8^+^T cell model. The age+CD8^+^T model provided greater net benefit than the age-only model across threshold probabilities of 0.30–0.60, indicating clinical net benefit in this range. When threshold probability exceeded 0.60, net benefit approached zero for both models, suggesting that very high thresholds should not be used as the sole diagnostic criterion. black dashed line:Treat all; black line: Treat none;green line: Age-only model;Red line: Age+CD8^+^T-cell model.
