# Supplementary material for: Age-dependent immune heterogeneity and the limited diagnostic utility of peripheral blood biomarkers in pediatric tuberculosis: a real-world cohort study with multidimensional model evaluation
Source: Front Immunol. 2026 Oct 7;17:1954622. doi: 10.3389/fimmu.2026.1954622 (PMC13682975; doi:10.3389/fimmu.2026.1954622)
Supplement: Supplementary file 1 [file DataSheet1.zip › Supplementary materials/Supplementary Table S1.docx]

## Supplementary Table S1 Pearson correlation matrix of candidate variables among all enrolled children (N = 280)

| **Variables** | **CD4⁺T** | **CD8⁺T** | **B cell** | **NK cell** | **LYM** | **Sex** | **Age** | **Vitamin D deficiency** | **MP co-infection** |
| --- | --- | --- | --- | --- | --- | --- | --- | --- | --- |
| CD4⁺T | 1 |  |  |  |  |  |  |  |  |
| CD8⁺T | 0.329** | 1 |  |  |  |  |  |  |  |
| B cell | 0.793** | 0.225** | 1 |  |  |  |  |  |  |
| NK cell | 0.569** | 0.391** | 0.418** | 1 |  |  |  |  |  |
| LYM | 0.736** | 0.408** | 0.692** | 0.500** | 1 |  |  |  |  |
| Sex | 0.084 | 0.087 | 0.118* | 0.155** | 0.133* | 1 |  |  |  |
| Age | -0.587** | -0.240** | -0.599** | -0.421** | -0.634** | -0.184** | 1 |  |  |
| Vitamin D deficiency | -0.201** | -0.164** | -0.155** | -0.195** | -0.168** | -0.142* | 0.227** | 1 |  |
| MP co-infection | -0.036 | -0.04 | -0.044 | -0.08 | -0.012 | 0.031 | 0.011 | 0.081 | 1 |

**Notes**: ** P < 0.01 (two-tailed); * P < 0.05 (two-tailed). CD4⁺T: CD4-positive T lymphocyte count; CD8⁺T: CD8-positive T lymphocyte count; B cell: B lymphocyte count; NK cell: natural killer cell count; LYM: total lymphocyte count; Sex: 0 = male, 1 = female; Vitamin D deficiency: 0 = normal vitamin D level, 1 = vitamin D deficiency; MP co-infection: 0 = without Mycoplasma pneumoniae co-infection, 1 = with MP co-infection.
