# Supplementary material for: Age-dependent immune heterogeneity and the limited diagnostic utility of peripheral blood biomarkers in pediatric tuberculosis: a real-world cohort study with multidimensional model evaluation
Source: Front Immunol. 2026 Oct 7;17:1954622. doi: 10.3389/fimmu.2026.1954622 (PMC13682975; doi:10.3389/fimmu.2026.1954622)
Supplement: Supplementary file 1 [file DataSheet1.zip › Supplementary materials/Supplementary Table S2.docx]

**Supplementary Table S2 Sensitivity analyses of the multivariate logistic regression model: substituting lymphocyte count with CD4⁺ T-cell count (Model 1) or vitamin D deficiency (Model 2).**

| Model | Variables | Adjusted OR | 95% CI | P-value |
| --- | --- | --- | --- | --- |
| **Model 1** | Gender | 1.137 | 0.658~1.963 | 0.654 |
|  | Age | 1.124 | 1.055~1.199 | <0.001 |
|  | CD4⁺ T cells | 1.000 | 0.999~1.000 | 0.165 |
|  | MP co-infection | 0.557 | 0.299~1.113 | 0.101 |
| **Model Diagnostics** |  |  |  |  |
| Hosmer-Lemeshow χ² (df=8) | - | 6.664, P=0.573 |  |  |
| Nagelkerke R² | - | 0.180 |  |  |
| **Model 2** | Gender | 1.047 | 0.603~1.818 | 0.870 |
|  | Age | 1.143 | 1.083~1.206 | <0.001 |
|  | Vitamin D deficiency | 2.096 | 1.083~4.057 | 0.028 |
|  | MP co-infection | 0.594 | 0.305~1.156 | 0.125 |
| **Model Diagnostics** |  |  |  |  |
| Hosmer-Lemeshow χ² (df=8) |  | 4.739, P=0.785 |  |  |
| Nagelkerke R² |  | 0.193 |  |  |

*Model 1 substituted LYM with CD4⁺ T-cell count; Model 2 substituted LYM with vitamin D deficiency (Yes vs. No). Both models were adjusted for age, sex, and MP co-infection. The adjusted ORs for age remained essentially unchanged in both sensitivity models (Model 1: OR ≈ 1.124, P < 0.001; Model 2: OR ≈ 1.143, P < 0.001), confirming the robustness of the primary finding.*
