# Supplementary material for: Age-dependent immune heterogeneity and the limited diagnostic utility of peripheral blood biomarkers in pediatric tuberculosis: a real-world cohort study with multidimensional model evaluation
Source: Front Immunol. 2026 Oct 7;17:1954622. doi: 10.3389/fimmu.2026.1954622 (PMC13682975; doi:10.3389/fimmu.2026.1954622)
Supplement: Supplementary file 1 [file DataSheet1.zip › Supplementary materials/Supplementary Table S3.docx]

**Supplementary Table S3 Comparison of immune-inflammatory parameters between MP-only controls and TB-MP co-infected cases**

| Indicators | MP-only controls (n=12) Median (IQR) | TB-MP co-infection (n=50) Median (IQR) |
| --- | --- | --- |
| CD4⁺ T（cells/μL） | 704.00（441.50–1435.50） | 637.00（497.75–847.50） |
| CD8⁺ T（cells/μL） | 583.50（335.25–788.25） | 402.50（355.75–541.25） |
| NK cells（cells/μL） | 173.00（111.25–222.00） | 156.00（91.25–252.50） |
| LYM（×10⁹/L） | 2.67（1.94–3.57） | 1.75（1.45–2.47） |
| Monocytes（×10⁹/L） | 0.45（0.28–0.90） | 0.40（0.29-0.51） |
