# Supplementary material for: Age-dependent immune heterogeneity and the limited diagnostic utility of peripheral blood biomarkers in pediatric tuberculosis: a real-world cohort study with multidimensional model evaluation
Source: Front Immunol. 2026 Oct 7;17:1954622. doi: 10.3389/fimmu.2026.1954622 (PMC13682975; doi:10.3389/fimmu.2026.1954622)
Supplement: Supplementary file 1 [file DataSheet1.zip › Supplementary materials/Supplementary Table S4.docx]

**Supplementary Table S4 Performance comparison between age-only and age+CD8⁺T models**

| **Metric** | **Model A (Age)** | **Model B (Age+CD8)** |  |
| --- | --- | --- | --- |
| **Sample** |  |  |  |
| N (events / non-events) | 280 (186 / 94) | 280 (186 / 94) |  |
| Discrimination |  |  |  |
| AUC (95% CI) | 0.686 (0.624–0.749) | 0.727 (0.668–0.786) |  |
| DeLong test P | — | 0.009 |  |
| **Calibration & Fit** |  |  |  |
| Hosmer–Lemeshow χ² / P | 14.61/0.067 | 10.57/0.228 |  |
| Brier score | 0.195 | 0.187 |  |
| McFadden R² | 0.094 | 0.127 |  |
| Likelihood-ratio test | — | χ² = 11.53, < 0.001 |  |
| **Coefficients** |  |  |  |
| Age (OR, P) | 1.156, <0.001 | 1.132, <0.001 |  |
| CD8 (OR, P) | — | 0.999, 0.011 |  |
| **Reclassification** |  |  |  |
|  | Estimate | 95% CI | P |
| Continuous NRI | 0.505 | 0.258–0.755 | < 0.001 |
| Event NRI⁺ | 0.419 | — | — |
| Non-event NRI- | 0.085 | — | — |
| IDI | 0.038 | 0.011–0.068 | 0.003 |

*Abbreviations: AUC, area under the curve; CI, confidence interval; IDI, Integrated Discrimination Improvement; NRI, Net Reclassification Improvement; OR, odds ratio.*
