# Supplementary material for: Age-dependent immune heterogeneity and the limited diagnostic utility of peripheral blood biomarkers in pediatric tuberculosis: a real-world cohort study with multidimensional model evaluation
Source: Front Immunol. 2026 Oct 7;17:1954622. doi: 10.3389/fimmu.2026.1954622 (PMC13682975; doi:10.3389/fimmu.2026.1954622)
Supplement: Supplementary file 1 [file DataSheet1.zip › Supplementary materials/Supplementary Table S5.docx]

**Supplementary Table S5 Diagnostic performance metrics of the age-only and age-combined predictive models**

| **Model** | **AUC (95% CI)** | **Optimal cutoff†** | **Sensitivity (%)** | **Specificity (%)** | **PPV (%)** | **NPV (%)** | **Youden index** |
| --- | --- | --- | --- | --- | --- | --- | --- |
| Age alone | 0.686 (0.618–0.755) | 8.290 | 0.828 | 0.500 | 0.766 | 0.595 | 0.328 |
| Age + NLR | 0.694 (0.626–0.762) | 0.606 | 0.785 | 0.564 | 0.781 | 0.570 | 0.349 |
| Age + SII | 0.690 (0.622–0.759) | 0.604 | 0.785 | 0.553 | 0.777 | 0.565 | 0.338 |
| Age + PLR | 0.711 (0.644–0.778) | 0.688 | 0.731 | 0.649 | 0.805 | 0.550 | 0.380 |
| Age + MP | 0.705 (0.639–0.770) | 0.677 | 0.726 | 0.585 | 0.776 | 0.519 | 0.311 |
| Age + CD8⁺ T | 0.727 (0.664–0.790) | 0.706 | 0.726 | 0.649 | 0.804 | 0.545 | 0.375 |
