# Supplementary material for: Age-dependent immune heterogeneity and the limited diagnostic utility of peripheral blood biomarkers in pediatric tuberculosis: a real-world cohort study with multidimensional model evaluation
Source: Front Immunol. 2026 Oct 7;17:1954622. doi: 10.3389/fimmu.2026.1954622 (PMC13682975; doi:10.3389/fimmu.2026.1954622)
Supplement: Supplementary file 1 [file DataSheet1.zip › Supplementary materials/Supplementary Table S6.docx]

**Supplementary Table S6 Age-matched sensitivity analysis: matching quality and immune parameter comparisons**

**Panel A. Age matching quality**

| Variable | Before matching | After matching |
| --- | --- | --- |
| TB / Control (n) | 186 / 94 | 83 / 83 |
| Age (years), mean ± SD | 12.71 ± 4.25 vs. 8.91 ± 5.87 | 10.33 ± 4.92 vs. 10.05 ± 5.27 |
| SMD | 0.744 | 0.056 |
| t-test P | <0.001 | 0.718 |

**Panel B. Immune parameter comparisons**

| Parameter | TB (n=83), median (IQR) | Control (n=83), median (IQR) | Matched P (Wilcoxon) | ≥12y stratum, matched P (n=41) |
| --- | --- | --- | --- | --- |
| CD4+T(cells/μL) | 665.0 (505.5–852.5) | 732.0 (563.0–965.0) | P = 0.113 | P = 0.569 |
| CD8+T(cells/μL) | 441.0 (346.0–553.5) | 548.0 (394.0–710.0) | P < 0.001 | P = 0.004 |
| NK(cells/μL) | 138.0 (91.5–233.0) | 213.0 (122.0–317.5) | P = 0.005 | P < 0.001 |
| WBC (×10⁹/L) | 6.79 (5.51–8.22) | 7.46 (5.79–10.11) | P = 0.013 | P = 0.315 |
| Lymphocytes (×10⁹/L) | 2.05 (1.49–2.80) | 2.43 (1.79–3.08) | P = 0.017 | P = 0.024 |
| PLR | 137.2 (104.5–197.9) | 116.8 (76.5–163.0) | P = 0.005 | P = 0.034 |
| B cells(cells/μL) | 311.0 (231.5–463.5) | 311.0 (214.0–473.5) | P = 0.663 | P = 0.385 |
| Monocytes (×10⁹/L) | 0.46 (0.34–0.60) | 0.47 (0.32–0.65) | P = 0.573 | P = 0.985 |
| Abbreviations: SMD, standardized mean difference; TB, tuberculosis; NK, natural killer cells; LYM, lymphocyte count; PLR, platelet-to-lymphocyte ratio; NLR, neutrophil-to-lymphocyte ratio; SII, systemic immune-inflammation index; MONO, monocyte count.  Before matching P values were derived from the overall cohort comparison using the Mann–Whitney U test (Table 2). †After matching P values were derived from the 1:1 age-matched cohort using the Wilcoxon signed-rank test; the ≥12 years paired subset P values were similarly derived from the matched subset. Age is presented as mean ± SD to calculate the SMD; an SMD <0.1 after matching indicates adequate balance between groups. | | | | |
