# Supplementary material for: Age-dependent immune heterogeneity and the limited diagnostic utility of peripheral blood biomarkers in pediatric tuberculosis: a real-world cohort study with multidimensional model evaluation
Source: Front Immunol. 2026 Oct 7;17:1954622. doi: 10.3389/fimmu.2026.1954622 (PMC13682975; doi:10.3389/fimmu.2026.1954622)
Supplement: Supplementary file 1 [file DataSheet1.zip › Supplementary materials/Supplementary Table S7.docx]

**Supplementary Table S7 Comparison of immune parameters between bacteriologically confirmed and clinically diagnosed TB cases (age-adjusted ANCOVA)**

| Indicator | Clinically diagnosed (n=118) Adjusted Mean (95% CI) | Bacteriologically confirmed (n=68) Adjusted Mean (95% CI) | P-value* (age-adjusted) | Partial η² |
| --- | --- | --- | --- | --- |
| CD4+T | 708.95(650.94-766.96) | 685.27(608.63-761.90) | 0.629 | 0.001 |
| CD8+T | 473.82(424.98-522.67) | 406.37(341.84-470.89) | 0.103 | 0.014 |
| NK cells | 176.32(154.58-198.07) | 164.25(135.52-192.98) | 0.512 | 0.002 |
| B cells | 357.54(314.36-400.72) | 334.89(277.85-391.94) | 0.535 | 0.002 |
| WBC | 6.88(6.37-7.40) | 6.52(5.84-7.20) | 0.403 | 0.004 |
| N | 4.12(3.65-4.59) | 3.83(3.21-4.46) | 0.466 | 0.003 |
| LYM | 2.13(1.97-2.29) | 1.98(1.78-2.19) | 0.256 | 0.007 |
| MONO | 0.45(0.40-0.50) | 0.51(0.453-0.574) | 0.105 | 0.014 |
| NLR | 2.28(1.959-2.596) | 2.452(2.031-2.873) | 0.518 | 0.002 |
| SII | 681.38(574.69-788.07) | 720.97(580.01-861.92) | 0.661 | 0.001 |
| PLR | 154.65(141.60-167.70) | 174.52(157.28-191.76) | 0.073 | 0.017 |
| *Adjusted for age using ANCOVA. Data are presented as estimated marginal means with 95% confidence intervals.  Abbreviations: ANCOVA, analysis of covariance; CI, confidence interval; LYM, lymphocyte; MONO, monocyte; NEUT, neutrophil; NLR, neutrophil-to-lymphocyte ratio; PLR, platelet-to-lymphocyte ratio; SII, systemic immune-inflammation index; TB, tuberculosis. | | | | |
